# Guillain-Barré syndrome and fulminant encephalomyelitis following Ad26.COV2.S vaccination: double jeopardy

**DOI:** 10.1186/s42466-022-00172-1

**Published:** 2022-02-08

**Authors:** Maria Ioanna Stefanou, Eleni Karachaliou, Maria Chondrogianni, Christos Moschovos, Eleni Bakola, Aikaterini Foska, Konstantinos Melanis, Elisabeth Andreadou, Konstantinos Voumvourakis, Matilda Papathanasiou, Eleni Boutati, Georgios Tsivgoulis

**Affiliations:** 1 Second Department of Neurology, National and Kapodistrian University of Athens, School of Medicine, “Attikon” University Hospital, Rimini 1, Chaidari, 12462 Athens, Greece; 2 First Department of Neurology, Aeginition Hospital, National and Kapodistrian University of Athens, Athens, Greece; 3 Second Department of Radiology, National and Kapodistrian University of Athens, School of Medicine, “Attikon” University Hospital, Athens, Greece; 4 Second Department of Internal Medicine, National and Kapodistrian University of Athens, School of Medicine, “Attikon” University Hospital, Athens, Greece

**Keywords:** Guillain-Barré syndrome, Encephalomyelitis, Transverse myelitis, Ad26.COV2.S, SARS-CoV-2 vaccine

## Abstract

This correspondence comments on a published article presenting a case of rhombencephalitis following SARS-CoV-2-vaccination with the mRNA vaccine BNT162b2 (Pfizer/BioNTech). We also present the case of a 47-year-old man who developed Guillain-Barré-syndrome and a fulminant encephalomyelitis 28 days after immunization with Ad26.COV2.S (Janssen/Johnson & Johnson). Based on the presented cases, we underscore the importance of clinical awareness for early recognition of overlapping neuroimmunological syndromes following vaccination against SARS-CoV-2. Additionally, we propose that that role of autoantibodies against angiotensin-converting enzyme 2 (ACE2) and the cell-surface receptor neuropilin-1, which mediate neurological manifestations of SARS-CoV-2, merit further investigation in patients presenting with neurological disorders following vaccination against SARS-CoV-2.

**Dear Editor**,

We have read with great interest the article by Walter and Kraemer [1] describing the case of a 30-year-old neurologist (one of the authors of the published report) presenting with rhombencephalitis following SARS-CoV-2-vaccination with the mRNA vaccine BNT162b2 (Pfizer/BioNTech). The authors report that the symptoms of rhombencephalitis, including left-sided facial paralysis, right-sided hypoglossal nerve palsy, and marked ataxia in all extremities, developed within 8 weeks after immunization with BNT162b2. Brain magnetic resonance imaging (MRI) studies revealed hyperintense lesions on fluid-attenuated inversion recovery (FLAIR) sequences, which were predominantly localized in the brainstem and cerebellum around the fourth ventricle and did not show contrast enhancement, while there was no evidence of spinal cord involvement. Besides a mild pleocytosis (cells:10/mm^3^), cerebrospinal fluid (CSF) analysis was normal. Based on the negative findings of an extensive diagnostic work-up, including infectious diseases, testing for oligoclonal bands (OCB), paraneoplastic, antineuronal, antinuclear, antineutrophil-cytoplasmic, myelin-oligodendrocyte-glycoprotein (MOG), antiganglioside and aquaporin-4 (AQP4) antibodies, a vaccine-induced autoimmune rhombencephalitis was suspected. The patient presented with gustatory function disorder that represents a frequent and predominantly immune-mediated manifestation of SARS-CoV-2 infection [2]. In view of this clinical presentation, the authors suggested that SARS-CoV-2-induced and COVID-vaccine-associated neurological adverse events are likely to share a common pathophysiological denominator.

In fact, although neurological adverse events following immunization against SARS-CoV-2 have been shown to be rare and less frequent in patients undergoing vaccination against SARS-CoV-2 compared to patients with Coronavirus Disease 2019 (COVID-19) [3–5], there are accumulating reports in the literature suggesting an association between COVID-vaccines and neuroimmunological complications similar to those observed in COVID-19 [6, 7]. Notably, neuroimmunological disorders, including Guillain-Barré-syndrome (GBS), acute transverse myelitis, acute disseminated encephalomyelitis, and Bell’s palsy, have been previously described following immunization with mRNA vaccines, including BNT162b2 and mRNA-1273 (Moderna), as well as adenovirus-vector COVID-19 vaccines, including ChAdOx1 nCOV-19 (AstraZeneca) and Ad26.COV2.S (Janssen/Johnson & Johnson) [6, 8–15]. Here, we report the case of a patient who developed GBS and a fulminant encephalomyelitis after vaccination with Ad26.COV2.S.

A previously healthy, 47-year-old man with unremarkable medical history presented with acral paresthesias and ascending flaccid paraparesis, 28 days after immunization with Ad26.COV2.S. GBS was diagnosed based on findings of (1) cytoalbuminologic dissociation (CSF protein: 5.6 g/l; cells: 2/mm^3^), (2) negative infectious work-up (including CSF Gram-staining, multiplexed CSF FilmArray® polymerase chain reaction [PCR] meningitis/encephalitis panel, SARS-CoV-2 PCR, and serological screening for syphilis, HIV, and HTLV1/2), (3) absence of systemic inflammatory markers (normal C-reactive protein levels and normal white blood cell count), and (4) electrophysiological evidence of prolonged F responses and abolished H-reflexes. The patient received treatment with intravenous immunoglobulin (2 gr/kg) with substantial improvement of his neurological symptoms. One week later, during the course of hospitalization, the patient deteriorated rapidly, developing a new T6-sensory-level and a severe tetraparesis (1/5 and 4/5 motor strength in the Medical Research Council [MRC] Scale in the lower and upper extremities, respectively). Upon clinical deterioration, magnetic resonance imaging (MRI) of the neuroaxis was performed, revealing striking neuroimaging findings (as shown in Fig. 1) compatible with a fulminant encephalomyelitis. Further antibody and OCB testing was not performed because the patient declined additional investigations. The patient received treatment with intravenous methylprednisolone (5gr) with gradual improvement of his motor and sensory symptoms. He was discharged with a mild residual paraparesis (4/5 and 5/5 muscle strength in the lower and upper extremities, respectively).

**Fig. 1:**
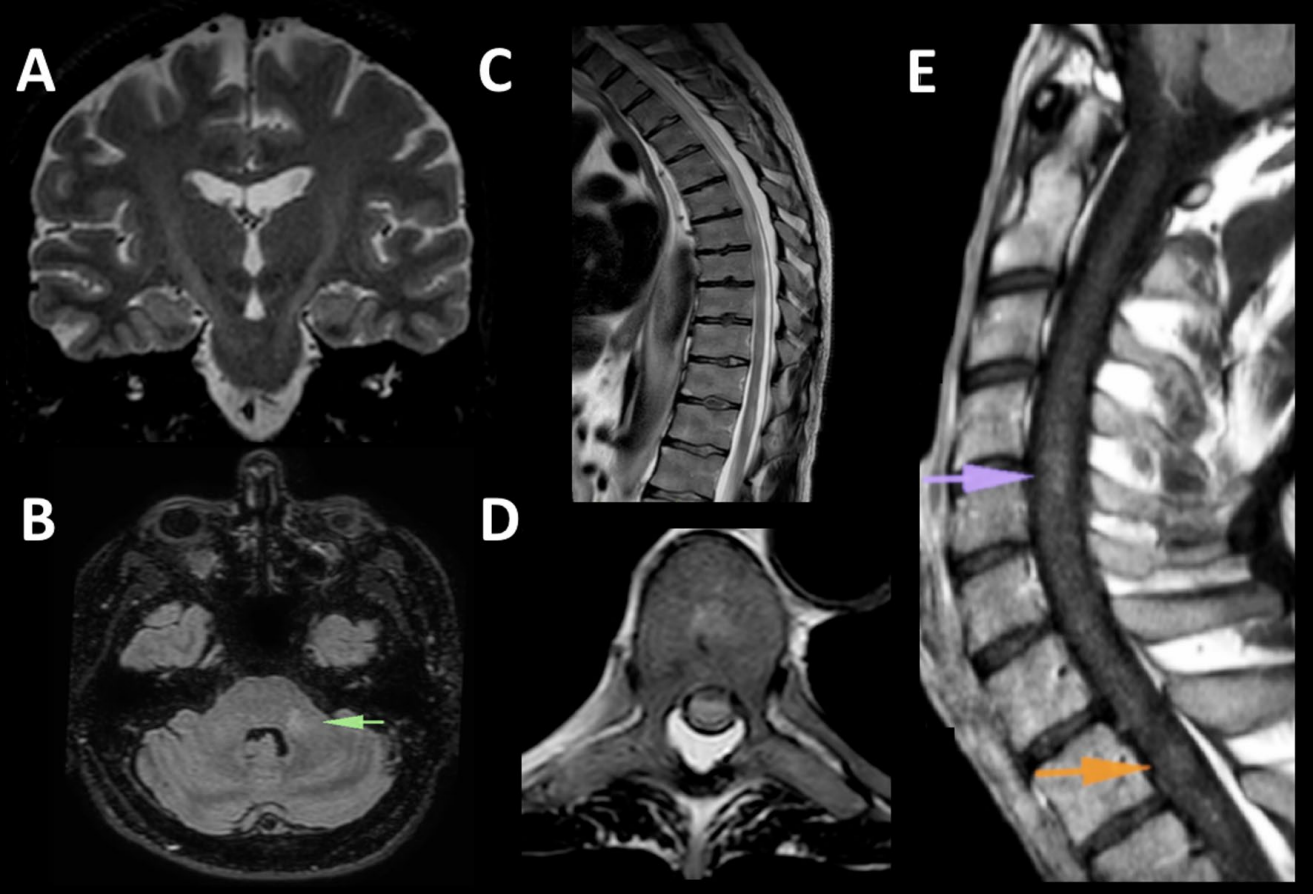
**A** Coronal T2-weighted MRI displays hyperintense signal along the corticospinal tracts bilaterally (“wine glass” sign). **B** On axial fluid attenuated inversion recovery (FLAIR), a hyperintense lesion is shown in the left middle cerebellar peduncle (green arrow). On brain MRI, no contrast enhancing lesions or lesions with diffusion restriction were depicted, while the optic nerves had normal appearance (images not shown). **C** Sagittal T2-weighted spine MRI shows longitudinally extensive thoracolumbar spinal cord lesions, with combined gray/white-matter involvement, affecting more than two-thirds of the thoracic spinal cord’s cross-sectional area on axial T2-weighted MRI sequence (**D**), findings compatible with transverse myelitis. **E** Sagittal T1-weighted gadolinium-enhanced MRI reveals contrast-enhancing lesions in the cervical and thoracic spinal cord (purple and orange arrows, respectively)

Although several reports have previously indicated an association between GBS and encephalomyelitis and Ad26.COV2.S vaccination [12, 16], the present case illustrates the importance of clinical awareness for early recognition for early-recognition of overlapping neuroimmunological syndromes after COVID-19 vaccination. Currently approved COVID-19 vaccines rely on the native viral spike protein (S) of SARS-CoV-2, which mediates viral entry by docking to the cell-surface angiotensin-converting enzyme 2 (ACE2) receptor for eliciting neutralizing antibodies [17]. These neutralizing antibodies were recently shown to compete with ACE2 for binding to the receptor-binding domain of SARS-CoV-2 [18]. It is thus, plausible that vaccine-induced SARS-CoV-2 antibodies could potentially exhibit an aberrant affinity for endogenous ACE2-receptors conferring an increased risk for autoimmunity that predominantly affects ACE2-receptor rich brain areas, similar to those affected in COVID-19 [19]. We therefore, find the neuroimaging findings of a predominantly periventricular lesion localization in the rhombencephalitis case reported by Walter and Kraemer [1] of particular interest, as ACE2-receptors are abundantly expressed in the ventricular system of the human brain [19]. Moreover, the hypothesis of a cross-reactivity between neutralizing antibodies and endogenous ACE2-receptors may explain an impaired function of ACE2-rich endothelial-cells of the cerebral microvasculature, which in turn could account for increased blood–brain permeability and demyelination noted in post-vaccination GBS and encephalomyelitis cases, respectively.

Furthermore, besides ACE2-receptors, additional host factors that facilitate SARS-CoV-2 cell-entry have been identified, including neuropilin-1, a cell-surface protein, that is abundantly expressed in the human brain, including the olfactory system. Neuropilin-1 holds a key role in neuronal development, endothelial function, and in the regulation of innate immune responses [20–24]. Previous research has linked alterations in the expression of neuropilin-1 to endothelial and blood–brain barrier dysfunction, as well as to aberrant neuroinflammatory responses in experimental autoimmune encephalomyelitis [25, 26]. In the setting of COVID-19, neuropilin-1 has been associated with the infectivity of SARS-CoV-2 and the severity of COVID-19-induced immunological responses [22, 24, 27]. To date, however, its potential role in neuroimmunological adverse events following COVID-19 vaccination remains to be elucidated. In conclusion, although further mechanisms may be implicated in neuroimmunological adverse events following COVID-19 vaccination, based on the experimental evidence provided herein, we suggest that the potential implication of autoantibodies against ACE2 and neuropilin-1 would be worth investigating in future cases presenting with neurological symptoms following vaccination against SARS-CoV-2 [28].

## Data Availability

Not applicable.
